# Supplementary material for: Reproductive Hormones Mediate Intestinal Microbiota Shifts during Estrus Synchronization in Grazing Simmental Cows
Source: Animals (Basel). 2022 Jul 7;12(14):1751. doi: 10.3390/ani12141751 (PMC9311722; doi:10.3390/ani12141751)
Supplement: Supplementary file 1 [file animals-12-01751-s001.zip › animals-1712760-supplementary.pdf]

Supplementary materials

Table S1. The chemical compositions of the herbage.

Itmes	Values
Dry matter (DM), %	34.7
Chemical composition, % DM	
Crude protein	16.9
Acid detergent fiber	37.6
Neutral detergent fiber	63.4
Ether extract	3.3
Ash	9.6
Non-fiber carbohydrate [1]	12.7
Starch	382
Calcium	0.61
Phosphorus	0.33

[1] Non-fiber carbohydrate is calculated as 100 − (crude protein + ether extract + ash + neutral detergent fiber).

Method:

Starter samples were analyzed for DM (method 930.15) [47], crude protein (method 976.05) [47], ether extract (method 920.39) [47], calcium (method 985.35) [47], and phosphorus (method 986.24) [47]. Starch was analyzed using a modified glucoamylase method [48]. Starch was hydrolyzed to free D-glucose, and the glucose concentration was then measured. Neutral detergent fiber (NDF) and acid detergent fiber (ADF) were determined according to methods of Van Soest et al. (1991) [49] with an Ankom 220 Fiber Analyzer (Ankom technology co., NY, USA). The NDF was analyzed with a heat stable α-amylase and sodium sulfite. Both NDF and ADF are expressed inclusive of residual ash.

Table S2. Significantly enriched predicted Level 3 KEGG pathways (relative abundance, %) on day 0 (D0), day 9 (D9), and day 12 (D12).

Pathway level1	Pathway level2	Pathway level2 Description	D0	D9	D12	SEM	*P*-value
Metabolism	Carbohydrate metabolism	Amino sugar and nucleotide sugar metabolism	1.3468[b]	1.3568[a]	1.3413[b]	0.00268	0.0008
Metabolism	Carbohydrate metabolism	Starch and sucrose metabolism	0.9915[a]	1.0018[a]	0.9684[b]	0.00522	0.0002
Metabolism	Carbohydrate metabolism	Fructose and mannose metabolism	0.8182[b]	0.8306[a]	0.8106[b]	0.00414	0.0056
Metabolism	Carbohydrate metabolism	Galactose metabolism	0.6893[b]	0.7008[a]	0.6788[c]	0.00266	< 0.0001
Metabolism	Carbohydrate metabolism	Butanoate metabolism	0.7025[a]	0.6900[b]	0.7078[a]	0.00360	0.0038
Metabolism	Carbohydrate metabolism	Pentose and glucuronate interconversions	0.4789[a]	0.4787[a]	0.4722[b]	0.00201	0.0347
Metabolism	Energy metabolism	Methane metabolism	1.3460[a]	1.3374[b]	1.3376[b]	0.00256	0.0349
Metabolism	Energy metabolism	Carbon fixation pathways in prokaryotes	1.0830[b]	1.0791[b]	1.0976[a]	0.00459	0.0173
Metabolism	Lipid metabolism	Glycerolipid metabolism	0.3465[ab]	0.3521[a]	0.3424[b]	0.00208	0.0078
Metabolism	Lipid metabolism	Arachidonic acid metabolism	0.0287[b]	0.0280[b]	0.0304[a]	0.00043	0.0011
Metabolism	Lipid metabolism	Steroid hormone biosynthesis	0.0082[b]	0.0087[b]	0.0101[a]	0.00042	0.0080
Metabolism	Lipid metabolism	Steroid biosynthesis	0.00082[b]	0.00118[b]	0.00184[a]	0.000186	0.0016
Metabolism	Lipid metabolism	Biosynthesis of unsaturated fatty acids	0.1177[b]	0.1183[b]	0.1219[a]	0.00090	0.0040

Metabolism	Lipid metabolism	Secondary bile acid biosynthesis	0.0269[a]	0.0279[a]	0.0254[b]	0.00049	0.0038
Metabolism	Lipid metabolism	Primary bile acid biosynthesis	0.0269[a]	0.0280[a]	0.0254[b]	0.00049	0.0023
Metabolism	Xenobiotics biodegradation and metabolism	Polycyclic aromatic hydrocarbon degradation	0.0987[b]	0.1006[a]	0.0984[b]	0.00054	0.0136
Metabolism	Biosynthesis of other secondary metabolites	Isoquinoline alkaloid biosynthesis	0.0577[ab]	0.0565[b]	0.0582[a]	0.00047	0.0442
Metabolism	Xenobiotics biodegradation and metabolism	Ethylbenzene degradation	0.0493[ab]	0.0485[b]	0.0505[a]	0.00053	0.0335
Metabolism	Xenobiotics biodegradation and metabolism	Atrazine degradation	0.0286[b]	0.0296[b]	0.0314[a]	0.00059	0.0069
Metabolism	Metabolism of terpenoids and polyketides	Biosynthesis of siderophore group nonribosomal peptides	0.0108[b]	0.0123[a]	0.0113[b]	0.00031	0.0076
Metabolism	Glycan biosynthesis and metabolism	Various types of N-glycan biosynthesis	0.00029[b]	0.00049[a]	0.00023[b]	0.000040	< 0.0001
Genetic Information Processing	Replication and repair	Non-homologous end-joining	0.00527[b]	0.00607[ab]	0.00660[a]	0.000354	0.0361
Genetic Information Processing	Transcription	Basal transcription factors	0.00066[ab]	0.00083[a]	0.00047[b]	0.00008	0.0125
Genetic Information Processing	Translation	Ribosome biogenesis in eukaryotes	0.0494[b]	0.0497[a]	0.0494[b]	0.00011	0.0448

Genetic Information Processing	Folding, sorting and degradation	Proteasome	0.04784^{b}	0.04813^{a}	0.04753^{c}	0.000101	0.0007
Organismal Systems	Endocrine system	Insulin signaling pathway	0.0667^{a}	0.0683^{a}	0.0646^{b}	0.00065	0.0013
Organismal Systems	Excretory system	Proximal tubule bicarbonate reclamation	0.0234^{ab}	0.0231^{b}	0.0248^{a}	0.00048	0.0347
Organismal Systems	Digestive system	Carbohydrate digestion and absorption	0.0054^{a}	0.0059^{a}	0.0044^{b}	0.00030	0.0044
Organismal Systems	Environmental adaptation	Circadian rhythm - plant	0.00029^{b}	0.00044^{ab}	0.00063^{a}	0.000071	0.0055

SEM = standard error of the means.

$^{a–c}$ Means without a common superscript within a row differ significantly ($P < 0.05$).

Table S3. The relationship of reproductive hormones and the biomarkers of the intestinal microbiota (uncorrelated results).

Item	GnRH	FSH	LH	PROG	E2	PRL	OT
p__Cyanobacteria	0.29	0.29	0.29	0.20	0.30	0.13	0.12
o__unclassified_c__Clostridia	0.22	0.06	0.16	0.11	0.16	0.05	0.14
f__Muribaculaceae	-0.08	0.03	-0.16	0.03	-0.23	0.02	0.01
f__Bacteroidaceae	-0.01	-0.07	-0.12	-0.16	0.11	0.18	-0.29
f__F082	-0.08	-0.18	-0.07	-0.29	0.12	-0.15	-0.11
f__unclassified_c__Clostridia	0.22	0.06	0.16	0.11	0.16	0.05	0.14
g__Prevotellaceae_UCG-004	-0.02	-0.07	-0.02	0.03	0.00	0.08	0.08
g__Bacteroides	-0.01	-0.07	-0.12	-0.16	0.11	0.18	-0.29
g__norank_f__Muribaculaceae	-0.09	0.16	-0.08	0.10	-0.22	-0.02	0.12
g__norank_f__Oscillospiraceae	-0.01	0.14	0.08	-0.26	0.12	0.11	-0.07
g__Alloprevotella	0.04	0.00	0.01	0.01	0.30	0.15	-0.15
g__norank_f__F082	-0.08	-0.18	-0.07	-0.29	0.12	-0.15	-0.11
g__Agathobacter	-0.05	-0.17	-0.16	-0.19	-0.21	0.00	-0.09
g__unclassified_c__Clostridia	0.22	0.06	0.16	0.11	0.16	0.05	0.14

g__UCG-002	0.08	0.18	0.21	0.08	0.22	0.24	0.16
p__Actinobacteriota	-0.19	**-0.36***	**-0.24**	**-0.13**	**-0.33***	**-0.10**	**0.05**
p__Verrucomicrobiota	0.25	**0.36***	**0.36***	**0.32***	**0.44****	**0.30**	**0.26**
p__Patescibacteria	-0.30	**-0.41****	**-0.20**	**-0.03**	**-0.30**	**-0.22**	**0.02**
o__Acidaminococcales	0.23	**0.27**	**0.22**	**0.08**	**0.38***	**0.06**	**0.01**
o__Christensenellales	-0.26	**0.03**	**0.11**	**0.15**	**0.16**	**0.08**	**0.40****
o__Saccharimonadales	-0.30	**-0.41****	**-0.20**	**-0.03**	**-0.30**	**-0.22**	**0.02**
o__Gastranaerophilales	0.29	**0.29**	**0.29**	**0.20**	**0.31***	**0.13**	**0.12**
o__Verrucomicrobiales	0.19	**0.34***	**0.31***	**0.28**	**0.41****	**0.31***	**0.27**
o__Coriobacteriales	-0.21	**-0.32***	**-0.31***	**-0.20**	**-0.27**	**-0.12**	**0.00**
c__Vampirivibrionia	0.19	**0.34***	**0.31***	**0.28**	**0.41****	**0.31***	**0.27**
c__Negativicutes	0.20	**0.25**	**0.21**	**0.07**	**0.38***	**0.06**	**0.01**
c__Coriobacteriia	-0.21	**-0.32***	**-0.31***	**-0.20**	**-0.27**	**-0.12**	**0.00**
c__Verrucomicrobiae	0.19	**0.34***	**0.31***	**0.28**	**0.41****	**0.31***	**0.27**
c__Saccharimonadia	-0.30	**-0.41****	**-0.20**	**-0.03**	**-0.30**	**-0.22**	**0.02**
f__Acidaminococcaceae	0.23	**0.27**	**0.22**	**0.08**	**0.38***	**0.06**	**0.01**

f__Christensenellaceae	-0.26	**0.03**	**0.11**	**0.15**	**0.16**	**0.08**	**0.40****
f__Hungateiclostridiaceae	-0.08	**-0.43****	**-0.21**	**-0.21**	**-0.35***	**-0.37***	**0.07**
f__norank_o__Gastranaerophilales	0.29	**0.29**	**0.29**	**0.20**	**0.31***	**0.13**	**0.12**
f__Akkermansiaceae	0.19	**0.34***	**0.31***	**0.28**	**0.41***	**0.31***	**0.27**
f__Atopobiaceae	-0.23	**-0.35***	**-0.35***	**-0.36***	**-0.38***	**-0.16**	**-0.07**
f__Saccharimonadaceae	-0.30	**-0.41****	**-0.20**	**-0.03**	**-0.30**	**-0.22**	**0.02**
g__Ruminococcus_torques_group	-0.22	**-0.42****	**-0.32***	**-0.29**	**-0.51**$^{\#}$	**-0.17**	**-0.22**
g__Phascolarctobacterium	0.24	**0.27**	**0.23**	**0.09**	**0.39***	**0.07**	**0.01**
g__Christensenellaceae_R-7_group	-0.27	**0.02**	**0.10**	**0.16**	**0.15**	**0.11**	**0.40****
g__norank_f__Ruminococcaceae	-0.19	**-0.16**	**-0.03**	**-0.05**	**-0.31***	**0.10**	**0.13**
g__Candidatus_Saccharimonas	-0.30	**-0.41****	**-0.20**	**-0.03**	**-0.30**	**-0.22**	**0.02**
g__norank_f__norank_o__Gastranaerophilales	0.29	**0.29**	**0.29**	**0.20**	**0.31***	**0.13**	**0.12**
g__Akkermansia	0.19	**0.34***	**0.31***	**0.28**	**0.41****	**0.31***	**0.27**
g__Saccharofermentans	-0.14	**-0.37***	**-0.30**	**-0.30**	**-0.44****	**-0.40****	**0.06**
g__Olsenella	-0.26	**-0.34***	**-0.35***	**-0.37***	**-0.40****	**-0.20**	**-0.10**

GnRH: gonadotropin-releasing hormone, FSH: follicle-stimulating hormone, LH: luteinizing hormone, PROG: progesterone, E2: Estrogen 2, PRL: prolactin, and OT: oxytocin.
